# S100B protein as abiomarker for prediction of brain death following head trauma

**Published:** 2012-11

**Authors:** Moslem Shakeri, Ali Meshkini, Ghaffar Shokouhi, Mohammad Asghari, Firooz Salehpoor, Atta Mahdkhah, Aidin Kazempoorazar

**Affiliations:** ^*a*^Department of Neurosurgery , Tabriz University of Medical Sciences, Tabriz, Iran.

## Abstract

**Background:**

S100B Protein is a biomarker that reflects post-trauma brain injury. In traumatic brain injury definite prediction of outcome of the patients is important goal. In this regard our study focuses on the S100B protein value in predicting brain death after head trauma.

**Methods:**

This was a cross-sectional study conducted on seventy-two patients (50 male and 22 female) aged 5-80 years (median 40 ± 17.72 years) with severe head trauma (GCS less than or equal to 8). For all patients, determination of GCS level and computed tomography (CT) scan were performed. Then, a single 5ml blood sample was obtained from each patient on admission, 48 hour later, and a week later or after brain death to determine the level of S100B protein.

**Results:**

Primary and last GCS of patients had a predictive value in determining brain death (p less than 0.0005). Furthermore, there was a significant correlation between GCS and level of S100B protein. Based on CT scan findings, there was a significant correlation between CT scan and S100B protein only after 48 hours post-trauma. Results of the present study demonstrate that the level of S100B protein at 48 hours after trauma can be used as a marker to predict brain death.

**Conclusions:**

In our study level of S100B protein at 48 hours after trauma was a good predictor of brain death. Based on the findings of the present study it can be concluded that a combined method incorporating the GCS test, CT scan, clinical symptoms and biomarkers can be a perfect tool for brain death prediction.

**Keywords:**

S100B protein, Head trauma, Brain death


					Volume 4, Suppl. 1 Nov 2012
Publisher: Kermanshah University of Medical Sciences
URL: http://www.jivresearch.org
Copyright:
Congress of Iranian Neurosurgeons, 14-16 November 2012, Kermanshah, Iran
Paper No. 11
S100B protein as abiomarker for prediction of brain death
following head trauma
Moslem Shakeri a
, Ali Meshkini a
, Ghaffar Shokouhi a
, Mohammad Asghari a
, Firooz Salehpoor a
,
Atta Mahdkhah a
,*, Aidin Kazempoorazar a
a
Department of Neurosurgery , Tabriz University of Medical Sciences, Tabriz, Iran.
Abstract:
Background: S100B Protein is a biomarker that reflects post-trauma brain injury. In traumatic brain injury definite
prediction of outcome of the patients is important goal. In this regard our study focuses on the S100B protein value in
predicting brain death after head trauma.
Methods: This was a cross-sectional study conducted on seventy-two patients (50 male and 22 female) aged 5-80
years (median 40  17.72 years) with severe head trauma (GCS less than or equal to 8). For all patients,
determination of GCS level and computed tomography (CT) scan were performed. Then, a single 5ml blood sample
was obtained from each patient on admission, 48 hour later, and a week later or after brain death to determine the
level of S100B protein.
Results: Primary and last GCS of patients had a predictive value in determining brain death (p less than 0.0005).
Furthermore, there was a significant correlation between GCS and level of S100B protein. Based on CT scan
findings, there was a significant correlation between CT scan and S100B protein only after 48 hours post-trauma.
Results of the present study demonstrate that the level of S100B protein at 48 hours after trauma can be used as a
marker to predict brain death.
Conclusions: In our study level of S100B protein at 48 hours after trauma was a good predictor of brain death.
Based on the findings of the present study it can be concluded that a combined method incorporating the GCS test,
CT scan, clinical symptoms and biomarkers can be a perfect tool for brain death prediction.
Keywords:
S100B protein, Head trauma, Brain death
* Corresponding Author at:
Atta Mahdkhah: Department of Neurosurgery, Tabriz University of Medical Sciences, Tabriz, Iran, Tel: 09144039122,
Email: mahdkhah@yahoo.com (Mahdkhah A.).

				

